# Supplementary material for: The Increased Densities, But Different Distributions, of Both C3 and S100A10 Immunopositive Astrocyte-Like Cells in Alzheimer’s Disease Brains Suggest Possible Roles for Both A1 and A2 Astrocytes in the Disease Pathogenesis
Source: Brain Sci. 2020 Jul 31;10(8):503. doi: 10.3390/brainsci10080503 (PMC7463428; doi:10.3390/brainsci10080503)
Supplement: Supplementary file 1 [file brainsci-10-00503-s001.pdf]

Supplementary Materials: The Increased Densities, but Different Distributions, of Both C3 and S100A10 Immunopositive Astrocyte-Like Cells in Alzheimer's Disease Brains Suggest Possible Roles for Both A1 and A2 Astrocytes in the Disease Pathogenesis

Andrew King, Boglarka Szekely, Eda Calapkulu, Hanan Ali, Francesca Rios, Shalmai Jones and Claire Troakes

Table S1. Details of antibodies used for immunoperoxidase analysis.

Antibody	Details	Supplier (Code)	Concentration
C3	Anti-C3d monoclonal rabbit	AbCam (ab 136916)	1:500
S100A10	Anti-S100A10 monoclonal mouse	Lifespan (LS-B3445)	1:500

Table S2. Details of antibodies used for immunofluorescence.

Antibody	Details	Supplier (Code)	Concentration
C3	Anti-C3d monoclonal rabbit	AbCam (ab136916)	1:200
S100A10	Anti-S100A10 monoclonal mouse	Lifespan (LS-B3445)	1:200
GFAP	Anti-GFAP polyclonal rabbit	DAKO (Z0334)	1:500
GFAP	Anti-GFAP monoclonal mouse	AbCam (ab4648)	1:1000

Table S3. Table showing details of the Control and Alzheimer's disease cases in the study including the relative densities of C3 and S100A10 immunopositive astrocyte-like glial cells in different cerebral areas.

	CASE	AGE	SEX	C3 Cerebrum Frontal	C3 Cerebrum Temporal	C3 Hippo	S100A10 Cerebrum Frontal	S100A10 Cerebrum Temporal	S100A10 Hippo	Other
C	BBN_4193	51	F	Frontal upp cx +/- Low cx ++ wm +			Frontal cx – wm ++ (pv)			
C	BBN_15612	44	M	Frontal upp cx +/- Low cx +++ wm +++			Frontal cx - wm +/- (pv)			Some oedema
C	BBN_17130	35	M	Frontal upp cx + Low cx + wm +++			Frontal upp cx +/- low cx +/- wm +/-			
C	BBN_16768	25	M		Temp upp cx +/- Low cx ++/+ wm +			Temp cx - wm +/-		Congen heart disease
C	BBN_16507	33	F	Frontal upp cx +/- Low cx +++ wm +++			Frontal cx – wm + (pv)			

C	BBN_16906	40	M			Hippo CA4 +/-			Hippo CA4 +	
C	BBN_21005	76	F	Frontal upp cx +/- Low cx +++ wm +++	Temp upp cx +/- Low cx + wm focal ++	Hippo CA4 +	Frontal cx – wm ++/+ (pv)	Temp upp cx + (pia) Low cx - wm ++/+	Hippo Ca4 +	
C	BBN002.32633	81	F	Frontal upp cx + Low cx +++ wm +++	Temp Upp cx +/- low cx+++ wm +++ (diffuse plaques +)	Hippo CA4 ++	Frontal upp cx +/- (pia) Low cx +/- wm +(pv)	Temp upp cx +/- (pia) Low cx - wm + (pv)	Hippo CA4 +/-	
C	BBN002.28350	74	M	Frontal upp cx +/- Low cx +++ wm +++	Temp upp cx – Low cx +++ wm +++	Hippo CA4 ++/+	Frontal cx – wm ++/+	Temp cx – wm +	Hippo Ca4 +	

				C3			S100A10			
	CASE	AGE	SEX	Cerebrum Frontal	Cerebrum Temporal	Hippo	Cerebrum Frontal	Cerebrum Temporal	Hippo	Other
C	BBN002.30171	86	F	Frontal upp cx +/- Low cx +++ wm +++	Temp upp cx – Low cx +++ wm +++	Hippo CA4 +++/++	Frontal upp cx +/- (pia) Low cx - wm +	Temp cx – wm ++/+	Hippo Ca4 ++	
C	BBN002.29089	81	M	Frontal upp cx + Low Cx ++	Temp upp cx + Low cx +++ wm +++	Hippo CA4 ++	Frontal upp cx + (pia) Low cx -	Temp cx – wm +	Hippo CA4 ++	

				wm ++/+ (focal)			wm + (pv)			
C	BBN002.32844	81	F	Frontal upp cx +/-<br>Low +++<br>Wm +++	Temp upp cx +/-<br>Low cx +++<br>wm +++	Hippo CA4 ++	Frontal cx-<br>wm ++/+ (pv)	Temp cx-<br>wm ++/+(pv)	Hippo CA4 +/-	
C	BBN_22594	77	F		Temp upp cx ++/+<br>Low cx +++<br>wm +++			Temp cx -<br>wm -		
C	BBN_16242	90	F	Frontal upp cx +/-<br>Low cx +++<br>wm +++	Temp upp cx +<br>Low cx +++<br>wm +++	Hippo CA4 N/A	Frontal cx –<br>Frontal wm -	Temp cx –<br>wm -	Hippo CA4 N/A	
C	BBN_16360	81	F	Frontal upp cx +/-<br>Low cx +<br>wm +	Temp upp cx +/-<br>Low cx +/-<br>wm ++ (focal)	Hippo CA4 +/-	Frontal upp cx +/-<br>Low cx -<br>wm + (pv)	Temp cx –<br>Wm + (pv)	Hippo CA4 -	Sepsis
C	BBN_16251	66	M	Frontal upp Cx +/-<br>Low cx +++<br>wm +++/++	Temp upp cx +/+<br>Low cx +++<br>wm +++	Hippo CA4 +	Frontal cx –<br>wm +/- (pv)	Temp cx –<br>Wm -	Hippo CA4 -	
C	BBN_16662	73	F	Frontal upp cx +	Temp upp cx ++/+	Hippo CA4 +	Frontal cx –	Temp upp cx +/-	Hippo CA4 –	

	CASE	AGE	SEX	C3 Cerebrum Frontal	C3 Cerebrum Temporal	C3 Hippo	S100A10 Cerebrum Frontal	S100A10 Cerebrum Temporal	S100A10 Hippo	Other
				Low cx +++ wm +++	Low cx +++ wm ++		wm +/- (pv)	Low cx +/- wm +/-		
C	BBN002.30882	77	F	Frontal upp cx - Low Cx + wm +	Temp upp cx – Low cx + wm ++/+	Hippo CA4 +	Frontal cx – Frontal wm + (pv)	Temp cx – Temp wm +(pv)	Hippo CA4 +/-	
C	BBN002.32844	81	F	Frontal upp Cx ++ Low Cx +++ wm +++	Temp upp cx + Low cx +++ wm +++	Hippo CA4 ++	Frontal upp cx +/- Low cx +/- wm + (pv)	Temp Cx – wm +/- (pv)	Hippo Ca4 +/-	COPD
C	BBN002.32817	97	F	Frontal upp cx +/- Low Cx + wm +/-	Temp upp cx – Low cx +/- wm – cx diff plaques +/-	Hippo CA4 +/- (diff plaques +/-)	Frontal upp Cx +/- Low cx +/- wm +/- cx diff plaques +/-	Temp upp cx – Low cx – wm +/- cx diff plaques +/-	Hippo CA4 –	
C	BBN002.32588	75	F	Frontal upp cx + Low cx ++ (focal) wm +++ (focal)	Temp upp cx – Low cx +++ wm +++	Hippo CA4 +	Frontal upp cx – Low cx – wm + (pv)	Temp upp cx – Low cx- wm +/- (pv)	Hippo CA4 +/-	

C	BBN002.35222	78	F	Frontal upp cx – Low cx ++ wm +++/++	Temp upp cx – Low cx +++ wm +++	Hippo CA4 ++	Frontal upp cx – Low cx – wm +	Temp upp cx – Low cx – wm +	Hippo CA4 -	

				C3			S100A10			
	**CASE**	**AGE**	**SEX**	**Cerebrum Frontal**	**Cerebrum Temporal**	**Hippo**	**Cerebrum Frontal**	**Cerebrum Temporal**	**Hippo**	**Other**
AD VI +LB	BBN_24557	86	F	Frontal upp cx + Low cx + wm +/- cx plaques ++ tangles	Temp upp cx + Low cx + wm + cx plaques +++ tangles	Hippo CA4 + plaques +++ tangles	Frontal upp cx +++ Low cx +++ wm +++ /++ cx plaques +++ tangles	Temp upp cx +++ Low cx +++ wm +++ cx plaques +++ tangles	Hippo CA4 +++ plaques +++ tangles	
AD VI + LB	BBN_24199	83	M	Frontal upp cx ++ Low cx +++ wm +++ cx plaques ++ tangles	Temp upp cx +++ Low Cx +++ wm +++ cx plaques +++	Hippo CA4 +++/++ Plaques +++	Frontal upp cx +++/++ Low cx + wm + cx plaques +++/++ tangles	Temp upp cx +++/++ Low cx +++/++ wm ++ cx plaques ++ tangles	Hippo CA4 ++ Plaques +	

AD V	BBN_16230	80	F	Frontal upp cx +/-<br>Low cx +/-<br>wm +<br>cx plaques +	Temp upp cx +/-<br>Low cx +/-<br>wm +/-<br>cx plaques +	Hippo Ca4 +<br>Plaques ++	Frontal upp cx +<br>Low cx ++/+<br>wm ++/+<br>cx plaques ++/+	Temp upp cx +++/++<br>Low cx +++/++<br>wm +<br>cx plaques +++/++	Hippo CA4 +<br>Plaques ++
AD VI	BBN_9824	71	F	Frontal upp cx ++<br>Low cx +++<br>wm +++<br>cx plaques +++	Temp upp cx +<br>Low cx +/-<br>wm +<br>cx plaques +++	Hippo CA4 ++<br>plaques +++	Frontal upp cx ++<br>Low cx ++<br>Wm +++<br>cx plaques +++	Temp upp cx ++<br>Low cx ++<br>wm +++<br>cx plaques +++	Hippo CA4 ++<br>plaques +++
AD V	BBN_9881	80	F	Frontal upp cx ++/+<br>Low cx +<br>wm ++<br>cx plaques ++	Temp upp cx ++/+<br>Low cx +<br>wm +<br>cx plaques +++	Hippo CA4 +<br>plaques +++	Frontal upp cx +++<br>Low cx ++<br>wm +++<br>cx plaques +++	Temp upp cx +++<br>Low cx ++<br>wm +++<br>cx plaques +++	Hippo CA4 +++<br>plaques +++
AD VI	BBN_9846	92	F	Frontal upp cx ++<br>Low cx +++<br>wm +++<br>cx plaques +++	Temp upp cx +++<br>Low cx +++<br>wm +++<br>Cx plaques +	Hippo CA4 +++<br>Plaques ++	Frontal upp cx ++<br>Low Cx ++/+<br>wm ++<br>cx plaques +++	Temp upp cx ++<br>Low cx ++<br>wm ++<br>Cx plaques +++	Hippo CA4 +++<br>plaques +++

**C3**	**S100A10**	**Other**

	CASE	AGE	SEX	Cerebrum Frontal	Cerebrum Temporal	Hippo	Cerebrum Frontal	Cerebrum Temporal	Hippo
AD VI	BBN_9898	80	M	Frontal upp cx ++/+ Low cx + wm + cx plaques +	Temp upp cx ++ Low cx + wm ++ cx plaques ++/+ tangles	Hippo CA4 + plaques +++	Frontal upp cx +++ Low cx ++/+ Wm ++/+ Cx plaques ++	Temp upp cx +++ Low cx +++ wm +++ cx plaques ++ tangles	Hippo CA4 ++/- Plaques +++
AD VI	BBN002.32856	74	F	Frontal upp cx +++/++ Low cx +++ wm ++ cx plaques +++	Temp upp cx ++ Low cx ++/+ wm +++ cx plaques +++	Hippo CA4 ++ Plaques ++	Frontal upp cx ++ low Cx ++ wm ++ cx plaques +++	Temp upp cx +++ low cx ++ wm ++ cx plaques +++ tangles	Hippo CA4 +++ plaques +++
AD VI	BBN00232829	78	F	Frontal upp Cx ++ Low cx ++ wm + cx plaques +++	Temp upp cx ++ Low cx ++ wm, ++ cx plaques ++	Hippo CA4 +/- Plaques +	Frontal upp cx ++ Low cx ++ wm + (pv) cx plaques +++	Temp upp cx ++ Low cx ++ wm ++/+ (pv) cx plaques +++	Hippo CA4 + plaques +
AD VI	BBN32545	75	F	Frontal upp cx +++ Low cx ++ wm +++ (focal) cx plaques +++	Temp upp cx ++ Low cx ++ wm ++ cx plaques +/-	Hippo CA4 ++/+ Plaques ++	Frontal upp cx +++ Low cx ++ wm ++ cx plaques +++	Temp upp cx ++ Low cx + wm ++ cx plaques ++	Hippo CA4 +++ plaques ++

AD VI	BBN002.32597	85	F	Frontal upp cx ++ Low cx ++/+ wm ++ cx plaques ++	Temp upp cx ++ Low cx + wm + cx plaques ++	Hippo CA4 ++ plaques ++	Frontal upp cx +++ Low cx ++ wm ++ cx plaques ++	Temp upp cx +++ Low cx ++/+ wm +++ cx plaques +++	Hippo CA4 ++ Plaques ++
AD VI	BBN002.33768	92	F	Frontal upp cx ++ Low cx ++ (focal) wm +++ cx plaques ++	Temp upp Cx + Low Cx ++ wm ++/+ cx plaques +/-	Hippo CA4 N/A	Frontal upp cx +++ Low cx +++/++ wm +++ cx plaques ++	Temp upp cx ++ Low cx ++ wm +++ Cx plaques ++	Hippo CA4 N/A

AD - Alzheimer's disease (followed by modified Braak (BNE) stage), C-Control, Congen-congenital, COPD-chronic obstructive pulmonary disease, cx-cortex, diff-diffuse, Hippo-Hippocampus, LB-Lewy Bodies , pia-astroctye(s) only seen in direct vicinity of pia, pv-perivascular only, temp-temoral, upp-upper, wm-white matter. Scores refer to density of astrocyte-like cells in particular areas unless specified otherwise: - negative, +/- only very infrequent, + infrequent, ++ moderate numbers/moderate density, +++ large numbers/high density.

Table S4. C3 immunopositive astrocyte-like cell counts in frontal upper and lower cortex and white matter.

C3	Controls		AD		Controls		AD		Controls		AD
Upper Cx				Lower Cx				Frontal wm			
BBN00232633		BBN00233768		BBN00232633		BBN00233768		BBN00232633		BBN00233768	
	1		10		31		4		53		28
	1		6		32		4		74		44
	2		14		26		3		60		30
	5		10		29		5		63		34
	6		8		24		6		51		32
	3		9		33		7		57		24
	5		5		33		14		50		25
	5		7		20		17		49		32
	4		8		26		13		42		22
	8		9		27		15		35		14
BBN00232588	4	BBN00232597	2	BBN00232588	6	BBN00232597	3	BBN00232588	39	BBN00232597	4

	2		2		0		4		32		12
	3		3		4		4		67		11
	2		1		7		2		42		13
	0		8		0		2		30		8
	1		3		6		5		28		5
	0		2		6		7		31		9
	0		5		6		1		24		2
	0		4		9		3		13		2
	2		3		11		1		19		9
BBN0023522	1	BBN32545	12	BBN0023522	5	BBN32545	4	BBN0023522	37	BBN32545	6
	0		5		16		2		32		20
	0		5		11		6		47		20
	1		4		9		3		22		15
	0		5		13		2		39		12
	1		4		13		1		33		22
	1		5		7		4		40		2
	0		3		11		5		23		19
	1		3		4		2		25		9
	0		4		21		4		20		2
BBN00232588	2	BBN00232856	2	BBN00232588	4	BBN00232856	19	BBN00232588	2	BBN00232856	34
	1		10		4		18		6		13
	2		4		5		19		5		14
	2		13		8		17		3		16
	2		14		4		13		3		16
	3		7		9		10		3		12
	2		14		4		19		2		13
	1		5		5		16		0		14
	2		14		7		21		2		13
	2		5		5		18		0		15
BBN00230882	0	BBN16230	16	BBN00230882	3	BBN16230	5	BBN00230882	5	BBN16230	4
	0		6		2		5		3		2
	1		7		4		8		6		7
	0		8		8		3		6		13
	0		8		5		4		3		9
	0		5		3		3		7		2
	0		10		2		2		2		2

	1		5		2		2		3		3
	1		5		1		2		3		3
	0		8		2		1		8		6
BBN16662	1	BBN9846	7	BBN16662	13	BBN9846	17	BBN16662	19	BBN9846	68
	1		7		14		27		16		89
	0		4		14		16		14		64
	1		2		8		16		17		67
	1		3		12		25		8		92
	2		4		14		17		25		73
	2		3		19		21		20		79
	2		4		16		18		14		67
	3		1		19		13		16		58
	4		1		16		17		12		55
BBN16251	3	BBN9824	4	BBN16251	5	BBN9824	25	BBN16251	13	BBN9824	65
	0		4		5		21		10		35
	3		5		5		33		9		64
	0		4		6		28		11		40
	1		8		11		30		15		16
	1		4		5		19		15		15
	2		9		5		23		10		16
	1		9		9		28		3		26
	0		4		3		29		5		33
	1		8		6		20		10		26
BBN16242	0	BBN9898	6	BBN16242	30	BBN9898	4	BBN16242	36	BBN9898	16
	1		8		32		5		53		26
	0		4		21		9		56		7
	0		6		16		2		39		11
	0		3		16		7		47		9
	1		2		20		6		53		9
	3		5		19		10		41		12
	0		6		16		8		36		9
	2		10		11		8		48		3
	1		6		20		9		37		8
SD	1.590916		3.393124		8.996123		8.673755		19.43567		22.42817
Mean	1.475		6.075		11.7375		10.8625		24.4625		23.2
SEM	0.17787		0.379363		1.005797		0.969755		2.172974		2.507546

H=84.02	H=0.852	H=0.475
P<0.001	P=0.3559	P=0.4906

Table S5. S100A10 immunopositive astrocyte like cell counts in frontal upper and lower cortex and white matter.

S100A10	Controls		AD	Controls			AD		controls		AD
Upper cx				Low Cx				Front wm			
BBN00232633		BBN00233768		BBN00232633		BBN00233768		BBN00232633		BBN00233768	
	0		6		0		5		1		17
	0		4		0		3		0		15
	0		4		0		5		0		15
	0		1		0		5		0		11
	0		5		0		7		1		18
	0		9		0		1		0		12
	0		5		0		2		1		11
	0		5		0		6		0		12
	1		3		0		6		0		9
	0		2		0		1		0		13
BBN00232588	0	BBN00232597	7	BBN00232588	0	BBN00232597	2	BBN00232588	0	BBN00232597	7
	0		3		1		1		0		7
	0		3		0		0		0		12
	0		3		0		0		1		11
	0		4		0		0		0		15
	0		6		1		2		0		10
	0		3		0		3		1		17
	0		6		0		1		0		10

	0		3		0		2		0		6
	0		1		1		4		0		2
BBN0023522	0	BBN32545	20	BBN0023522	0	BBN32545	3	BBN0023522	1	BBN32545	1
	0		9		0		3		0		2
	0		7		0		0		1		1
	0		7		0		3		0		1
	0		9		0		3		0		5
	0		7		0		1		0		2
	0		8		0		2		0		3
	0		6		0		4		2		2
	0		4		0		2		0		2
	0		8		0		0		0		2
BBN0023258 8	1	BBN0023285 6	2	BBN0023258 8	2	BBN0023285 6	3	BBN0023258 8	1	BBN0023285 6	6
	0		4		1		3		3		4
	0		0		0		2		0		6
	0		2		0		2		0		7
	0		1		0		3		0		4
	0		2		0		2		1		4
	1		2		1		2		0		6
	0		1		0		0		2		13
	0		6		1		1		0		3
	1		4		0		2		0		5
BBN0023088 2	0	BBN16230	3	BBN0023088 2	0	BBN16230	6	BBN0023088 2	0	BBN16230	8
	0		2		0		4		0		11
	1		2		0		4		0		7

	0		3		0		9		0		9
	0		3		0		10		0		5
	0		0		0		7		1		7
	0		4		0		13		0		20
	0		2		1		2		1		16
	0		2		0		3		2		11
	0		3		0		1		1		16
BBN16662	0	BBN9846	3	BBN16662	0	BBN9846	1	BBN16662	0	BBN9846	5
	0		1		0		2		0		7
	0		1		0		1		0		7
	0		1		0		1		0		5
	0		3		0		2		2		4
	0		1		0		3		0		5
	0		4		0		3		0		3
	0		15		0		1		0		8
	0		4		0		4		0		7
	0		9		0		1		0		5
BBN16251	0	BBN9824	8	BBN16251	0	BBN9824	3	BBN16251	1	BBN9824	9
	0		5		0		0		0		10
	0		3		0		4		1		6
	0		1		0		6		0		9
	0		4		0		4		0		13
	0		6		0		1		0		9
	0		1		0		2		1		7
	0		7		0		1		0		6
	0		2		0		1		0		5
	0		5		0		1		0		6

BBN16242	0	BBN9898	9	BBN16242	0	BBN9898	3	BBN16242	0	BBN9898	3
	0		7		0		3		0		4
	0		5		0		5		0		3
	1		7		0		3		0		1
	0		10		0		1		0		3
	0		2		0		1		0		4
	0		16		0		3		0		2
	1		10		0		3		0		0
	0		4		0		3		0		3
	0		2		0		2		0		4
SD	0.284349		3.57239<br>8		0.35556<br>2		2.31721		0.63195<br>5		4.68224<br>5
Mean	0.0875		4.65		0.1125		2.8125		0.325		7.275
SEM	0.031791		0.39940<br>6		0.03975<br>3		0.25907<br>2		0.07065<br>5		0.52349<br>1
		H=110.335				H=16.202				H=91.5	
		P<0.001				P<0.001				P< 0.001	

Table S6. AD cases - Comparison of C3 and S100 astrocyte-like cell counts in upper and lower frontal cortex and white matter.

**Upper cx AD**	**AD**	**lower cx AD**	**AD**	**Frontal wm AD**	**AD**
S100A10	C3	S100A10	C3	S100A10	C3
BBN00233768	BBN00233768	BBN00233768	BBN00233768	BBN00233768	BBN00233768
6	10	5	4	17	28
4	6	3	4	15	44
4	14	5	3	15	30
1	10	5	5	11	34
5	8	7	6	18	32

	9		9		1		7		12		24
	5		5		2		14		11		25
	5		7		6		17		12		32
	3		8		6		13		9		22
	2		9		1		15		13		14
BBN00232597	7	BBN00232597	2	BBN00232597	2	BBN00232597	3	BBN00232597	7	BBN00232597	4
	3		2		1		4		7		12
	3		3		0		4		12		11
	3		1		0		2		11		13
	4		8		0		2		15		8
	6		3		2		5		10		5
	3		2		3		7		17		9
	6		5		1		1		10		2
	3		4		2		3		6		2
	1		3		4		1		2		9
BBN32545	20	BBN32545	12	BBN32545	3	BBN32545	4	BBN32545	1	BBN32545	6
	9		5		3		2		2		20
	7		5		0		6		1		20
	7		4		3		3		1		15
	9		5		3		2		5		12
	7		4		1		1		2		22
	8		5		2		4		3		2
	6		3		4		5		2		19
	4		3		2		2		2		9
	8		4		0		4		2		2
BBN00232856	2	BBN00232856	2	BBN00232856	3	BBN00232856	19	BBN00232856	6	BBN00232856	34
	4		10		3		18		4		13

	0		4		2		19		6		14
	2		13		2		17		7		16
	1		14		3		13		4		16
	2		7		2		10		4		12
	2		14		2		19		6		13
	1		5		0		16		13		14
	6		14		1		21		3		13
	4		5		2		18		5		15
BBN16230	3	BBN16230	16	BBN16230	6	BBN16230	5	BBN16230	8	BBN16230	4
	2		6		4		5		11		2
	2		7		4		8		7		7
	3		8		9		3		9		13
	3		8		10		4		5		9
	0		5		7		3		7		2
	4		10		13		2		20		2
	2		5		2		2		16		3
	2		5		3		2		11		3
	3		8		1		1		16		6
BBN9846	3	BBN9846	7	BBN9846	1	BBN9846	17	BBN9846	5	BBN9846	68
	1		7		2		27		7		89
	1		4		1		16		7		64
	1		2		1		16		5		67
	3		3		2		25		4		92
	1		4		3		17		5		73
	4		3		3		21		3		79
	15		4		1		18		8		67
	4		1		4		13		7		58

	9		1		1		17		5		55
BBN9824	8	BBN9824	4	BBN9824	3	BBN9824	25	BBN9824	9	BBN9824	65
	5		4		0		21		10		35
	3		5		4		33		6		64
	1		4		6		28		9		40
	4		8		4		30		13		16
	6		4		1		19		9		15
	1		9		2		23		7		16
	7		9		1		28		6		26
	2		4		1		29		5		33
	5		8		1		20		6		26
BBN9898	9	BBN9898	6	BBN9898	3	BBN9898	4	BBN9898	3	BBN9898	16
	7		8		3		5		4		26
	5		4		5		9		3		7
	7		6		3		2		1		11
	10		3		1		7		3		9
	2		2		1		6		4		9
	16		5		3		10		2		12
	10		6		3		8		0		9
	4		10		3		8		3		3
	2		6		2		9		4		8
sd	3.572398		3.393124		2.31721		8.673755		4.682245		22.42817
Mean	4.65		6.075		2.8125		10.8625		7.275		23.2
SEM	0.399406		0.379363		0.259072		0.969755		0.523491		2.507546
		H=9.804				H=50288				H=34.956	
		P=0.0017				P<0.001				P<0.001	

Table S7. Controls- Comparison of C3 and S100 astrocyte-like cell counts in upper and lower frontal cortex and white matter.

S100A10	Controls	C3		S100A10	Controls		C3		S100A10	controls	C3	
Upper cx				Lower Cx					Frontal wm			
BBN00232633		BBN00232633			BBN00232633		BBN00232633		BBN00232633		BBN00232633	
	0		1			0		31		1		53
	0		1			0		32		0		74
	0		2			0		26		0		60
	0		5			0		29		0		63
	0		6			0		24		1		51
	0		3			0		33		0		57
	0		5			0		33		1		50
	0		5			0		20		0		49
	1		4			0		26		0		42
	0		8			0		27		0		35
BBN00232588	0	BBN00232588	4		BBN00232588	0	BBN00232588	6	BBN00232588	0	BBN00232588	39
	0		2			1		0		0		32
	0		3			0		4		0		67
	0		2			0		7		1		42
	0		0			0		0		0		30
	0		1			1		6		0		28
	0		0			0		6		1		31
	0		0			0		6		0		24
	0		0			0		9		0		13
	0		2			1		11		0		19
BBN0023522	0	BBN0023522	1		BBN0023522	0	BBN0023522	5	BBN0023522	1	BBN0023522	37
	0		0			0		16		0		32
	0		0			0		11		1		47
	0		1			0		9		0		22
	0		0			0		13		0		39
	0		1			0		13		0		33
	0		1			0		7		0		40
	0		0			0		11		2		23
	0		1			0		4		0		25
	0		0			0		21		0		20

BBN00232588	1	BBN00232588	2	BBN00232588	2	BBN00232588	4	BBN00232588	1	BBN00232588	2
	0		1		1		4		3		6
	0		2		0		5		0		5
	0		2		0		8		0		3
	0		2		0		4		0		3
	0		3		0		9		1		3
	1		2		1		4		0		2
	0		1		0		5		2		0
	0		2		1		7		0		2
	1		2		0		5		0		0
BBN00230882	0	BBN00230882	0	BBN00230882	0	BBN00230882	3	BBN00230882	0	BBN00230882	5
	0		0		0		2		0		3
	1		1		0		4		0		6
	0		0		0		8		0		6
	0		0		0		5		0		3
	0		0		0		3		1		7
	0		0		0		2		0		2
	0		1		1		2		1		3
	0		1		0		1		2		3
	0		0		0		2		1		8
BBN16662	0	BBN16662	1	BBN16662	0	BBN16662	13	BBN16662	0	BBN16662	19
	0		1		0		14		0		16
	0		0		0		14		0		14
	0		1		0		8		0		17
	0		1		0		12		2		8
	0		2		0		14		0		25
	0		2		0		19		0		20
	0		2		0		16		0		14
	0		3		0		19		0		16
	0		4		0		16		0		12
BBN16251	0	BBN16251	3	BBN16251	0	BBN16251	5	BBN16251	1	BBN16251	13
	0		0		0		5		0		10
	0		3		0		5		1		9

	0		0		0		6		0		11
	0		1		0		11		0		15
	0		1		0		5		0		15
	0		2		0		5		1		10
	0		1		0		9		0		3
	0		0		0		3		0		5
	0		1		0		6		0		10
BBN16242	0	BBN16<br>242	0	BBN16242	0	BBN16242	30	BBN16242	0	BBN16<br>242	36
	0		1		0		32		0		53
	0		0		0		21		0		56
	1		0		0		16		0		39
	0		0		0		16		0		47
	0		1		0		20		0		53
	0		3		0		19		0		41
	1		0		0		16		0		36
	0		2		0		11		0		48
	0		1		0		20		0		37
SD	0.284349		1.5909<br>16		0.3555<br>62		8.99612<br>3		0.6319<br>55		19.43567
Mean	0.0875		1.475		0.1125		11.7375		0.325		24.4625
SEM	0.031791		0.1778<br>7		0.0397<br>53		1.00579<br>7		0.0706<br>55		2.172974
		H=<br>47.921				H=112.315				H=110.765	
		P<0.001				P<0.001				P<0.00<br>1	
